# The impact of RNA sequence library construction protocols on transcriptomic profiling of leukemia

**DOI:** 10.1186/s12864-017-4039-1

**Published:** 2017-08-17

**Authors:** Ashwini Kumar, Matti Kankainen, Alun Parsons, Olli Kallioniemi, Pirkko Mattila, Caroline A. Heckman

**Affiliations:** 1 Institute for Molecular Medicine Finland (FIMM), Helsinki Institute of Life Science, University of Helsinki, P.O. Box 20, Tukholmankatu 8, FI-00014 Helsinki, Finland; 2 Medical and Clinical Genetics, University of Helsinki and Helsinki University Hospital, Helsinki, Finland; 3 Science for Life Laboratory, Karolinska Institutet, Solna, Sweden; 4 Finnish Red Cross Blood Service, Kivihaantie 7, Helsinki, Finland

**Keywords:** RNA-sequencing, Hematological malignancies, Library preparation, Ribo-depletion, Poly-A selection

## Abstract

**Background:**

RNA sequencing (RNA-seq) has become an indispensable tool to identify disease associated transcriptional profiles and determine the molecular underpinnings of diseases. However, the broad adaptation of the methodology into the clinic is still hampered by inconsistent results from different RNA-seq protocols and involves further evaluation of its analytical reliability using patient samples. Here, we applied two commonly used RNA-seq library preparation protocols to samples from acute leukemia patients to understand how poly-A-tailed mRNA selection (PA) and ribo-depletion (RD) based RNA-seq library preparation protocols affect gene fusion detection, variant calling, and gene expression profiling.

**Results:**

Overall, the protocols produced similar results with consistent outcomes. Nevertheless, the PA protocol was more efficient in quantifying expression of leukemia marker genes and showed better performance in the expression-based classification of leukemia. Independent qRT-PCR experiments verified that the PA protocol better represented total RNA compared to the RD protocol. In contrast, the RD protocol detected a higher number of non-coding RNA features and had better alignment efficiency. The RD protocol also recovered more known fusion-gene events, although variability was seen in fusion gene predictions.

**Conclusion:**

The overall findings provide a framework for the use of RNA-seq in a precision medicine setting with limited number of samples and suggest that selection of the library preparation protocol should be based on the objectives of the analysis.

**Electronic supplementary material:**

The online version of this article (doi:10.1186/s12864-017-4039-1) contains supplementary material, which is available to authorized users.

## Background

RNA sequencing (RNA-seq) has become an important technology in the comprehensive analysis of disease transcriptomes and holds great promise for clinical applications including disease diagnosis, therapeutic selection, and precision medicine strategies [1–4]. The technique has been particularly insightful in understanding the pathogenesis and classification of leukemia [5, 6]. For example, it has enabled identification of a wide variety of clinically relevant predictive expression biomarkers [4, 7], fusion-genes and recurrent mutations [8, 9], expressed variants [5], and alternative splicing events [10] in different leukemia types. However, as a relatively new technology, sample preparation protocols and data analysis methods are still in their infancy and require further testing before RNA-seq can be translated to standard clinical practice [2].

Generation of a sequencing library for RNA-seq analysis is a complex, multi-step process and a potential source of significant variation [11, 12]. This process is most commonly carried out using poly-A-tailed mRNA selection (PA) or rRNA depletion (RD) to eliminate rRNAs that are naturally abundant in the sample and which would otherwise dominate the sequence data [13, 14]. However, both of these mainstream methods have their own advantages and limitations. For example, recent studies have noted that the RD protocol captures a wide repertoire of transcripts [15, 16] and works efficiently with degraded RNA [12, 15]. The high number of intron mapping reads in RD datasets may also be advantageous in understanding pre-mRNA dynamics and the post-transcriptional impact of microRNAs [17]. In contrast, Li et al. have reported PA libraries to contain less intronic reads than RD libraries [12] thereby offering a more cost-effective solution for gene expression studies [18]. The PA method also appears to outperform the RD protocol in detecting differentially expressed genes [15, 18]. However, the assessments of different RNA-seq library preparation protocols have mostly relied on non-clinical samples [16, 18–20], emphasizing the need for systematic comparison of library preparation protocols using patient samples. To address this need, our comparative analysis provides recommendations for the application of RNA-seq in clinical or pre-clinical settings with a limited number of samples.

In this study, we tested the performance of PA and RD protocols on samples from acute myeloid leukemia (AML) and acute lymphoblastic leukemia (ALL) patients in a personalized medicine setting. For each patient sample we generated two RNA-seq libraries using the PA and/or RD protocols. We then assessed the effects of the two different protocols on i) expression of protein coding and non-coding RNAs, ii) differential gene expression analysis, iii) pathway analysis, iv) fusion gene detection, and v) expressed variant calling. In addition, we measured the variability introduced by library preparation in technical replicates along with biological replicates and developed metrics applicable in routine medical practice and other small-*n* settings by integrating RNA-seq data with variant, biomarker, and ex vivo drug sensitivity and resistance testing (DSRT) data. Our analyses showed that PA and RD protocols produced consistent results and that patient heterogeneity represented the largest source of variation. However, the RD method captured more transcriptome features whereas PA outperformed the RD protocol in detecting differentially expressed genes and leukemic markers. Importantly, some of the observed discrepancies were clinically relevant and therefore selection of the protocol is a crucial step in clinical decision-making. Our results are directly relevant for researchers and healthcare professionals aiming to apply RNA-seq in a precision medicine setting to examine transcriptomes of hematological diseases for clinical assessment and indicate that the selection of the library preparation protocol should be guided by the study objectives.

## Methods

### Patient material

The Helsinki University Hospital Ethics Committee has approved the study and collection of samples (permit numbers 239/13/03/00/2010, 303/13/03/01/2011). Bone marrow (BM) aspirates from two AML and two ALL patients were collected after signed informed consent and with protocols in accordance with the Declaration of Helsinki. Mononuclear cells (MNCs) were isolated by density gradient separation from the BM of the patients (Ficoll-Paque PREMIUM; GE Healthcare; Little Chalfont, Buckinghamshire, UK).

### mRNA purification and library construction

Total RNA was extracted from MNCs using the Qiagen miRNeasy kit (Qiagen, Hilden, Germany). The kit is capable of isolating all types of RNA from a minimal amount of starting material. Short RNAs (< 200 nt) present in the total RNA were removed prior to preparation of the RNA-seq libraries. Next, RNA was quantified using the Qubit fluorometer (Thermo Fisher, Carlsbad, CA, USA), while the quality of the RNA samples was measured using a Bioanalyzer instrument and RNA nano chips (Agilent, Santa Clara, CA, USA). For the PA protocol, 2.5-5 microgram of total RNA from the ALL 542 and AML 800 samples was then subjected to oligo(dT) selection using the Dynabeads® mRNA Purification Kit (Thermo Fisher) as per the manufacturer’s instructions. The RD protocol was carried out from 2.5-5 microgram of total RNA from ALL 542, ALL 668, AML 800 and AML 1867 samples using the Ribo-Zero™ rRNA Removal Kit (Epicentre, Madison, WI, USA) as per the manufacturer’s instructions. After PA or RD selection, the samples were purified using Agencourt AMPure XP SPRI beads (Beckman Coulter, Brea, CA, USA) to remove chemical contaminants and short RNAs less than 200 nt in length.

PA and RD samples were further reverse transcribed to double stranded cDNA using the SuperScript Double-Stranded cDNA Synthesis Kit (Thermo Fisher). Random hexamers (New England BioLabs, Ipschwich, MA, USA) were used for priming the first strand synthesis reaction. Samples were prepared for RNA-seq using Illumina compatible Epicentre Nextera™ technology. After limited cycle PCR the RNA-seq libraries were size selected (350–700 bp fragments) in 2% agarose gel followed by purification with the QIAquick gel extraction kit (Qiagen).

### RNA sequencing

Each transcriptome was loaded to occupy one third of the lane capacity in the flow cell. The cBot-2 system and TruSeq PE Cluster Kit v3 (Illumina, San Diego, CA, USA) were used for cluster generation, and TruSeq SBS Kit v3-HS reagent kit and HiSeq2000 instrument (Illumina, San Diego, CA, USA) was used to generate paired 100-bp reads according to the manufacturer’s instructions. Nextera Read Primers 1 and 2 as well as Nextera Index Read Primer (Illumina) were used for paired-end sequencing and index read sequencing, respectively.

### Data analysis

Detailed descriptions of the data analysis methods, tools and information of used published data are provided in Additional file 1.

### Real-time quantitative reverse transcription-PCR (qRT-PCR)

Total RNA was extracted from two patients (ALL 542 and AML 800) and four breast cancer cell lines BT-474, MCF-7, KPL-4 and SKBR3 with on column DNase treatment. The RNA was quantified using the Qubit fluorometer. For each sample, the RNA was divided into three fractions for total RNA and PA and RD processing. PA capture was carried out using Dynabeads mRNA Purification Kit (Thermo Fisher). RD was performed with Ribo-Zero Magnetic Gold Kit (Epicentre). The cDNA was synthesized using SuperScript III Reverse Transcriptase (Thermo Fisher). The qRT-PCR reactions were prepared using 10 ng of cDNA from each cell line or patient sample plus the iQ SYBR Green Super Mix (Bio-Rad, Hercules, CA, USA), and reactions run on the CFX96 Real Time System instrument (Bio-Rad). Normalized fold expression values were calculated by the ΔΔC_t_ method using *B2M*, *GAPDH*, *PGK1*, and *RPLP0* as reference genes and total RNA as control [21]. The primer sequences are listed in Additional file 2: Table S8.

### PCR and Sanger sequencing

To validate the suspected fusion genes standard PCR was performed on cDNA from the ALL 542 and AML 800 samples. The cDNA was synthesized from total RNA using SuperScript III Reverse Transcriptase (Thermo Fisher). Primers were designed for *ST3GAL1-NDAG1*, *MCM4-PRKDC*, *HBB-B2M*, *PQLC1-CTDP1, NCL-NR4A1* (Additional file 2: Table S8). The cDNA was amplified with Taq polymerase and using the T Professional thermocycler (Biometra, Göttingen, Germany). No template and *GAPDH* were included as negative and positive controls, respectively. PCR products were run on a 3% agarose gel, stained with GelRed Nucleic Acid Stain (Biotium, Fremont, CA, USA) and visualized on a standard UV trans illuminator. The DNA fragment for the *HBB-B2M* fusion gene was excised from the gel, cleaned using the NucleoSpin Gel and PCR Clean-up kit (Macherey-Nagel, Düren, Germany), and quantified using the Qubit dsDNA HS kit (Thermo Fisher). 4.5 ng of the fragment was used for Sanger sequencing using both forward and reverse primers of the *HBB-B2M* fusion gene and standard sequencing protocols.

### Statistical analysis

Statistical analyses were performed with R version 3.3.1 (2016-06-21) and Prism software version 6.0 (GraphPad Software, San Diego, CA, USA). In the qRT-PCR analysis, two-tailed Student’s T-test was used to analyze gene expression and *P*-values <0.05 were considered as statistically significant. Statistical dependence between two variables was calculated by Spearman’s rank, Pearson’s correlation analysis and hypergeometric distribution as implemented in R.

## Results

### Overview of the study design

**Fig. 1:**
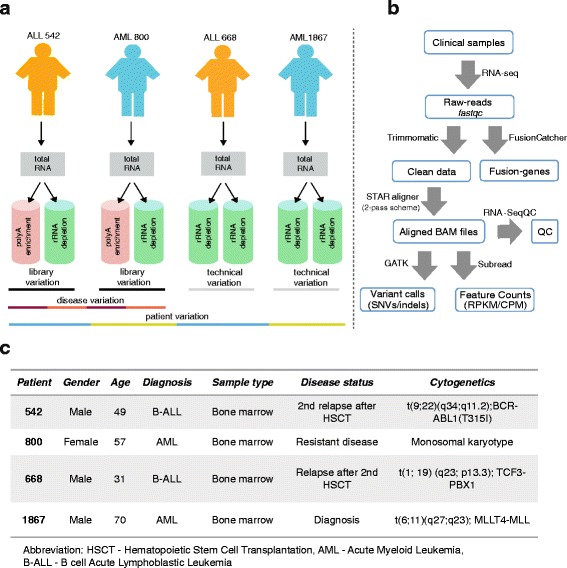
Experimental design and data analysis workflow of the study. **a** Bone marrow aspirates were collected from two ALL and two AML patient samples. Total RNA was isolated and used for RNA-seq library preparation by RD and PA protocols from the same RNA source. Altogether, two PA enriched and six RD libraries were constructed. The ALL 542 and AML 800 samples were used to analyze variation between library construction protocols and ALL 668 and AML 1867 samples used for technical variation comparison. **b** The flow chart shows the steps and tools used for RNA-seq data analysis. **c** Clinical characteristics of the leukemia patients

To determine the relative merits of two mainstream RNA-seq protocols in a setting with a limited number of clinical samples, we prepared technical, experimental, and biological replicate RNA-seq libraries (Fig. 1a). Altogether eight libraries were prepared from samples collected from two AML (patients 800 and 1867) and two ALL (patients 542 and 668) patients. These included two pairs of experimental replicates constructed from the same total RNA source (800 and 542) and two pairs of technical replicate libraries (1867 and 668). All libraries were prepared from high-quality RNA (RNA integrity number ≥ 8.2) and were subjected to paired-end sequencing using an Illumina HiSeq instrument. Analytical comparisons were focused on features relevant for clinical application, therapy optimization, and disease diagnosis, including transcript quantification, gene expression patterns, differential gene expression, and fusion gene discovery. The data analysis steps, such as quality check, reference genome alignment, fusion gene identification, variant calling, and gene expression quantifications are summarized in Fig. 1b. Clinical details of each patient are shown in Fig. 1c.

### Reads and read mapping statistics

**Fig. 2:**
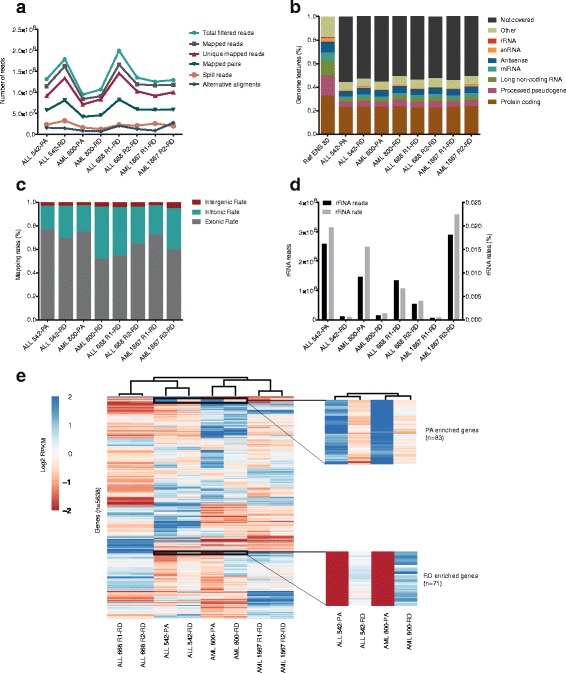
Comparative transcriptome analysis. **a** Alignment and mapping statistics, Y-axis representing the total number of reads sequenced from each library. More reads were captured in the RD as compared to the PA enriched libraries. **b** Bio-detection plot, Y-axis representing the percentage of genomic features. The bars correspond to the percentage of each biotype in the human reference genome (Ensembl 80). **c** The percentage of mapped reads to intragenic, intronic and exonic regions, Y-axis shows the read mapping rates. The replicate samples with the RD library showed much higher variation in the number of exonic reads compared to samples from different libraries. **d** Y-axis on the left and Y-axis on the right represent the number and rates of rRNA mapped reads, respectively. The RD method is more effective at rRNA removal as compared to the PA protocol. **e** The heatmap represents the patient, disease and library specific variations. Genes with log2 RPKM >2 and CV >20 were selected for hierarchical clustering. Clustering was performed with both genes and samples using a Euclidean distance and complete linkage method

We generated on average 146.9 million 94-base-pair reads (73.5 paired-end read-pairs) for each sample. The base quality along these reads was uniform and high, with a median quality score > 30 for all bases. The estimated average fragment sizes were also similar (280 bp in PA and 294 bp in RD), further suggesting a high level of technical similarity between the different libraries. From the raw reads, an average of 137.5 million reads (89.9-97.2%) passed quality processing and on average 121.3 million (78.8-88.8%) mapped to the reference genome (Fig. 2a). The libraries also had a comparable percentage (95.0-97.3%) of reads mapping to known human genes. However, the PA protocol provided higher fractions of exon-mapping reads (75.2-76.9%) compared to the RD libraries (52.0-72.6%). Conversely, reads mapping to intronic regions increased from an average of 21.0% in PA to 33.8% in RD (Fig. 2c). No major differences were seen in normalized gene body read count distributions (Additional file 3: Figure S1), revealing an accumulation of reads in the midpoint of the transcripts.

### Expression landscape

We observed in total 30,205 features in the PA libraries as compared to 32,830 features detected in their matched RD counterparts using an RPKM (reads per kilobase per million) value of 0.125 as a threshold for minimum expression [22] (Additional file 3: Figure S2). While rather similar sets of features were captured, some differences were observed in specific transcript classes, such as processed pseudogene (7.82% to 7.03%), lincRNA (5.35% to 4.67%), and snRNA (6.99% to 6.54%) elements, as presented in a biodetection plot (Fig. 2b, Additional file 2: Table S1). Overall, the RD protocol detected 20.8 to 26.3% more of these features compared to PA. Antisense and miRNA features were also called at greater level by the RD protocol, while rRNA elements were enriched in the PA protocol. Notably, discrepancies in the calling of lincRNA, miRNA, and antisense genes resulted mainly from protocol differences, given the similar levels of these types of calls across the technical replicates (Fig. 2b). We also observed subtle but biologically intriguing variations in the detection of protein coding gene transcripts. For example, a total of 1380 protein-coding genes were called discordantly between the matched PA and RD libraries at RPKM threshold of 0.125 (Additional file 3: Figure S3), which is close to twice that observed between technical replicates (patient 668 and 1867; Additional file 3: Figure S4). Further analysis of discordantly identified protein coding transcripts revealed most of these to be attributable to RNA preparation (Additional file 2: Table S2). On one hand, 55 of a total of 71 histone genes (hypergeometric distribution *p*-value <0.05) were overlooked by the PA library protocol at RPKM value of 0.125. On the other hand, protein-coding genes relevant to cancer such as *TGF-β1* mediating the activation of TGF-β/SMAD signaling pathway in ALL cells [23], *BCL3*, a proto-oncogene candidate associated with B-cell leukemia [24], and *BRD4,* which is associated with transcriptional deregulation in leukemia [25] were overlooked by the RD protocol at this threshold.

### rRNA removal efficiency

To compare the rRNA depletion efficiency of the RD and PA protocols, the fraction of reads aligned to known human rRNA sequences in each library was quantified by aligning reads to the rRNA precursor sequences with the RNA-SeQC software [26]. Rather unexpectedly, the PA libraries exhibited higher rRNA mapping read rates than RD libraries (1.8% vs. 0.6%; Fig. 2d). However, the rRNA mapping rates varied highly (2.24% to 0.04% and 0.67% to 0.48%) even between technical replicate libraries.

### Reproducibility of transcript abundances

Assessment of the concordance of protein-coding transcript abundances was made by measuring the correlation of RPKMs between different datasets (Additional file 3: Figure S6). We found a high level of concordance between the RPKMs of matched PA and RD samples (Spearman rho >0.95) and technical replicates (Spearman rho >0.98) in agreement with previously published results [18]. Since RPKM values between protocol-matched biological replicates (Spearman rho >0.91) and between AML and ALL samples (Spearman rho >0.88) were less correlated, patient heterogeneity appeared to represent the largest source of variation in these data. The hierarchical clustering of protein-coding transcripts with RPKM >4 and coefficient of variation >20 also corroborated the above findings and revealed that clustering was driven by disease type and patient rather than library type (Fig. 2e).

### Accuracy in expression-based leukemia classification

**Fig. 3:**
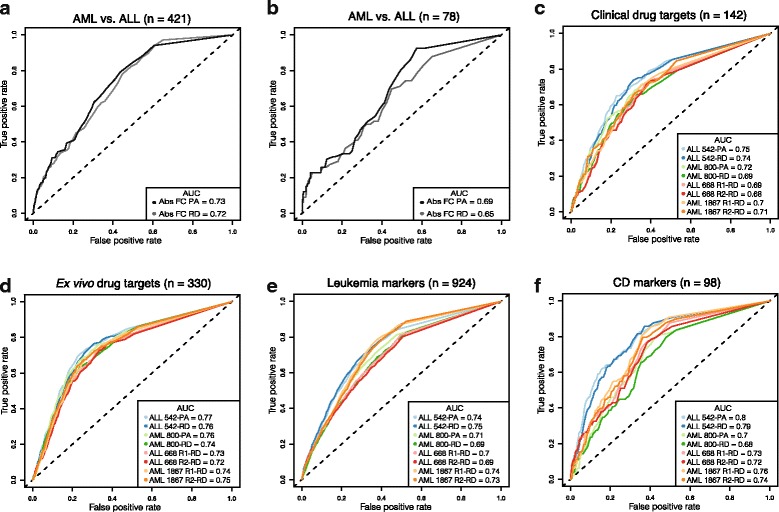
Receiver operating characteristics (ROC) analysis. **a, b** ROC curves of differentially expressed genes between AML and ALL, the plot shows higher AUC with the PA method. **c** Genes were selected from the literature based on putative targets of 17-oncology drugs used in clinic for the AML and ALL treatment. **d** The gene targets of 47 approved and investigational oncology compounds, which had high efficacy towards primary AML and ALL samples based on in-house ex vivo drug testing data. **e** Leukemia specific marker genes from microarray based published studies. **f** Cluster of differentiation (CD) marker genes. The PA protocol provided constantly higher RPKM values for the target genes in all comparisons

RNA-seq is often used to identify disease associated transcriptional profiles and for expression-based disease classification. To assess how the PA and RD protocols perform in these popular tasks, the utility of protocols to classify known leukemia marker and indicator genes was measured by plotting the true positive rate (sensitivity) against the false positive rate (100-specificity) at different fold-changes and RPKM cut-offs to compute the area under the receiver operating characteristic (ROC) curve (AUC). Importantly, both protocols distinguished target genes from the remaining human transcriptome well in this test, which produces robust results even in the lack of replicates. The PA protocol, however, systematically surpassed the RD protocol and generated 1-4% better AUC measurements. For example, it achieved a 1% larger AUC as compared to the RD protocol in detecting fold-differences in expression in a set of 421 AML and ALL indicator genes reported by Haferlach and colleagues [27] (Fig. 3a), and 4% larger AUC in detecting a set of 78 genes differentially expressed between AML and ALL in four independent microarray studies (Fig. 3b) [28–31]. The PA protocol also provided consistently higher RPKM values for the target genes for 17 clinically approved drugs used for AML and ALL treatment (Fig. 3c), ex vivo experimental drug candidates that were either highly sensitive or resistant in each patient case (in-house drug sensitivity and resistance testing experiments) [32, 33] (Fig. 3d), and for the leukemia-specific marker genes (Fig. 3e and f). The list and details of clinically approved drugs, experimental drugs and gene targets used for ROC analyses are available in Additional file 1 and Additional file 2: Table S3.

### Independent validation of RD and PA expression estimates

**Fig. 4:**
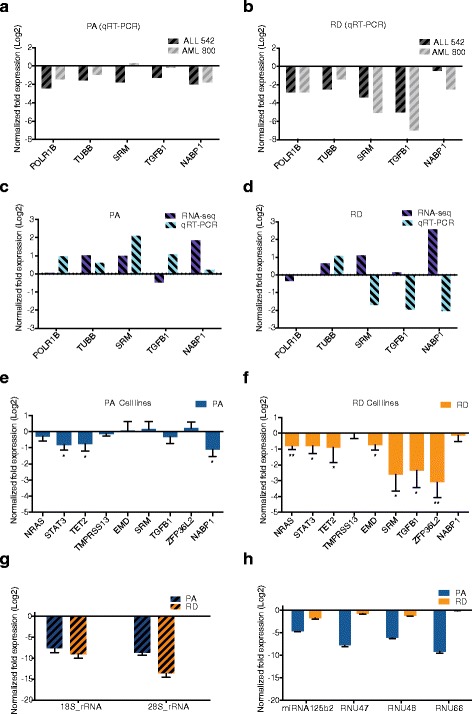
Validation by quantitative reverse transcription PCR (qRT-PCR). Normalized fold expression values represented on the X-axis were calculated by the ΔΔC_t_ method using four reference genes (*B2M*, *GAPDH*, *PGK1*, *RPLP0*) and then normalized to total RNA as control. Error bars represent the standard error of the mean ΔC_t_ values across all cell lines. Student’s T-test showing significant differences (*P* < 0.05). **a**, **b** The qRT-PCR log2-fold change expression of 5 genes in two leukemia patient samples (ALL 542 and AML 800) using PA and RD protocols compared to total RNA, showing the PA protocol prepared RNA is closer to total RNA. **c**, **d** The log2-fold expression of AML compared to ALL samples from both PA and RD libraries demonstrates the higher agreement of RNA-seq (log2 RPKM) and qRT-PCR (log2-fold expression) expression estimates in the PA than RD protocol. **e**, **f** The bar plots show the log2-fold change expression of 9 genes in four breast cancer cell lines MCF7, SKBR3, KPL1, BT474 using PA and RD protocols compared to total RNA. **g**, **h** The log2-fold expression of 18 s and 28 s rRNA compared to the expression in total RNA in ALL 542 and AML 800 patient samples, depicting higher efficiency of the RD protocol at removing rRNAs compared to the PA protocol. The log2-fold expression of 4 non-coding RNA in two cell lines (MCF7 and SKBR3) showing higher efficiency for the non-coding RNAs of the RD protocol compared to the PA protocol

To further evaluate the impact of library preparation on mRNA expression estimates, we prepared i) total, ii) PA, and iii) RD processed RNA isolates from two leukemia patient samples and four breast cancer cell lines and measured the expression of a set of oncogenes by qRT-PCR. Interestingly, analysis of the expression of five oncogenes in the patient samples showed that the RD protocol captured target mRNAs less efficiently than the PA protocol (Fig. 4a and b). While only one gene, *NABP1,* was significantly depleted after PA processing, three genes (*POLR1B, SRM,* and *TGFB1*) were all depleted in the RNA from the RD protocol (Additional file 3: Figure S7). Next, expression changes of these five genes between AML and ALL within the protocols were computed and compared to those derived using RNA-seq, validating our finding that the PA protocol suits better for differential gene expression analysis (Fig. 4c and d). Similarly, we observed that the target genes were under detected in the RD library in an independent set of four human breast cancer cell lines when compared to total RNA (Fig. 4e and f). For the PA library prepared RNA, we had similar observations, although the difference between total RNA was less than that seen in patient samples. Importantly, individual gene expression assessment in the cell lines showed that out of 9 tested genes, only *STAT3, NABP1* and *TET2* were significantly depleted in the PA enriched library (Fig. 4e), whereas *NRAS, STAT3, TET2, EMD, SRM, TGFB1,* and *ZFP36L2* all showed a significant difference between the RD library and total RNA (Fig. 4f). Finally, we observed that the RD method efficiently depleted rRNA compared to the PA protocol confirming our findings from the RNA-seq data (Fig. 4g). As expected, three small nucleolar RNAs and one miRNA in two breast cancer cell lines (MCF7, SKBR3) were not captured as efficiently by the PA compared to the RD method (Fig. 4h), indicating that the PA protocol may overlook non-coding transcripts and confirming also this aspect of our results.

### Fusion gene detection

**Fig. 5:**
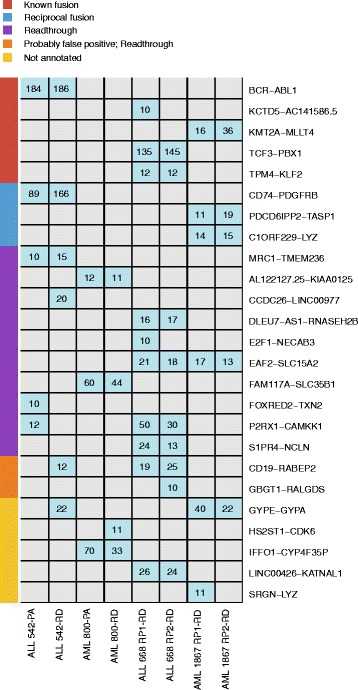
Fusion gene detection. The figure shows fusion genes reported by the FusionCatcher tool in five different categories including known fusion, not annotated fusion, reciprocal fusion, readthrough, and probably false positive. Only fusions supported by ≥10 spanning reads were selected. The number of supported spanning reads is shown in the heatmap for each fusion gene

The detection of genome rearrangements and transcriptional abnormalities resulting in in-frame expressed fusion genes plays a major role in the diagnosis and treatment of many hematological and other cancers [8, 34]. However, routine clinical testing is currently limited to the detection of a few well-known fusions while RNA-seq could allow means for their global interrogation [2]. To evaluate the efficiency of the PA and RD protocols in detecting fusion genes, the FusionCatcher [35] software tool was applied to the datasets. Altogether, the analysis reported 25 high quality (supported by ≥ 10 spanning reads; Fig. 5) and 102 other fusion gene candidates (Additional file 3: Figure S8) that were categorized into five types. Most fusion calls were read-throughs (48.8%) followed by putative fusions (25.1%), probably false positive read-throughs (10.2%), known fusions (7.8%), already known fusion read-throughs (3.9%), and reciprocal fusions (3.9%) (Additional file 3: Figure S8). Of these, the known fusions genes represented the clinically most important candidates with well-known roles in leukemia pathogenesis. Included in this category was the *BCR-ABL1* in-frame fusion gene that was supported by >180 spanning pair-end reads in both ALL 542 libraries, two in-frame fusions *TCF3-PBX1* (135 and 145 spanning reads) and *TPM4-KLF2* (12 spanning reads in each replicate) that were detected in both ALL 668 libraries, and *KMT2A* − *MLLT4* (16 and 36 spanning reads) that was discovered in both AML 1867 libraries (Fig. 5). In contrast to the high-quality detections, more variance was seen across detections with <10 read pairs. For example, the known fusion *ST3GAL1 − NDRG1* (CDS-truncated) was detected only in ALL 542-PA (5 spanning reads), the *MCM4-PRKDC* (UTR-intronic) only in ALL 542-RD (3 spanning reads), *PQLC1-CTDP1* (6 spanning reads) and *ARL17A-KANSL1* (4 spanning reads) only in AML 800-RD (Additional file 3: Figure S8). Interestingly, lower quality fusion gene detection among technical replicates also varied notably. For example, only one of the replicates supported the presence of the known fusion genes *KCTD5-AC141586.5* (10 spanning reads) and *OAZ1-KLF2* (4 spanning reads) in ALL 668, while the *TPM4* − *KLF2* (12 spanning reads) was identified only in one AML 1867-RD library (Additional file 3: Figure S8), indicating that low coverage may indeed impair fusion gene detection.

To confirm some of the low confidence fusion genes, we tested the presence of five fusion partners with discordant fusion detections in two samples (AML 800 and ALL 542) using a standard PCR assay. PCR amplification of *ST3GAL1-NDRG1* (5 spanning reads in ALL 542-PA), *MCM4-PRKDC* (3 spanning reads in ALL 542-RD), *PQLC1-CTDP1* (6 spanning reads in AML 800-RD), *NCL-NR4A1* (3 spanning reads in AML 800-PA) and *HBB-B2M* (8 spanning reads in ALL 542-RD) failed to detect the fusion gene, except *HBB-B2M* in ALL 542-RD. However, Sanger sequencing did not confirm the *HBB-B2M* fusion, which could be due to low abundance of the fusion in the sample or false positive prediction by the FusionCatcher tool (Additional file 2: Table S9).

### Variant detection

**Fig. 6:**
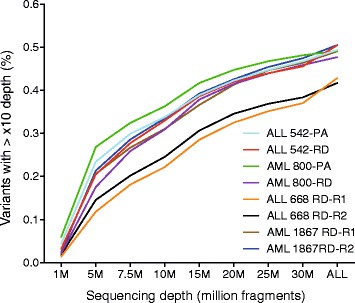
Coverage efficiency as a fraction of number of reads. The percentage of targeted bases covered at >10× depth. The PA protocol captures higher fractions of target bases

Variant detection based on RNA-seq data provides an efficient means to detect sequence variation in those genes that are expressed in the sample [36]. However, the suitability of the two mainstream RNA-seq library preparation protocols on variant discovery has not yet been addressed thoroughly using patient samples. To understand the potential of each protocol to characterize variants associated with AML and ALL, we extracted the genomic coordinates of all nonsense (743 bp analyzed), missense (12,782 bp analyzed), frameshift deletion (2044 bp analyzed) and frameshift insertion (1047 bp analyzed) AML and ALL variants from the COSMIC database [37] and computed their coverage efficiency as a function of sequencing depth (Fig. 6). Similar to the results observed in gene expression analyses, the PA method outperformed the RD method and captured a higher amount of target bases at all read count and depth cut-offs (Fig. 6, Additional file 3: Figure S5). The most profound difference was at low read counts (from 1 M to 10 M), which is best explained by the lower rate of intron mapped reads in the PA libraries (Fig. 2c) and high fraction of exonic variants in the test data. For example, the RNA-seq data originating from the PA protocol covered around 25, 31 and 35% of target regions with at least 15× depth, capturing roughly 6, 4, and 3% more AML and ALL associated variants than the RD protocol, respectively. At higher read counts we observed more marginal differences, with both protocols capturing >42% of the target bases (Fig. 6). Higher read depths also increased the consistency (Additional file 2: Table S4). For example, a higher level of concordance between matched RD and PA libraries was observed at ≥15× (Phi correlation >0.82) than ≥5× (Phi correlation >0.69) threshold. Overall, the level of concordance was higher between technical replicates (Phi correlation >0.86 at ≥15×) and matched RD and PA libraries (Phi correlation >0.82 at ≥15×) than between biological replicates (Phi correlation >0.75 at ≥15×).

In addition to the capture efficiency analysis, we performed variant effect annotation and filtering analysis on discovered variants. On average, 131,623 variants and 293 filtered variants were discovered in the process (Additional file 2: Table S5). Among these were several affecting one of the top 20 mutated genes for AML or ALL (Additional file 2: Table S6). For example, ALL 542-PA and ALL 542-RD had mutations in *CREBBP* (p.L161 fs) and *ABL1* (p.T315I; present in 73 samples in COSMIC) genes, while AML 800-PA and AML 800-RD supported a mutation in the *TP53* gene (p.R273C; present in 142 COSMIC samples). In addition to these three variants supported by both library preparation protocols, AML 800-PA revealed the presence of a mutation in the *TET2* gene (p.V1949 fs). The library replicates detected mutations in *DNMT3A*, *NRAS*, *TET2*, *BCOR*, and *CREBBP* genes, indicating that variant discovery had high technical reproducibility and concordance.

### Effect of library preparation on pathway enrichment analysis

**Fig. 7:**
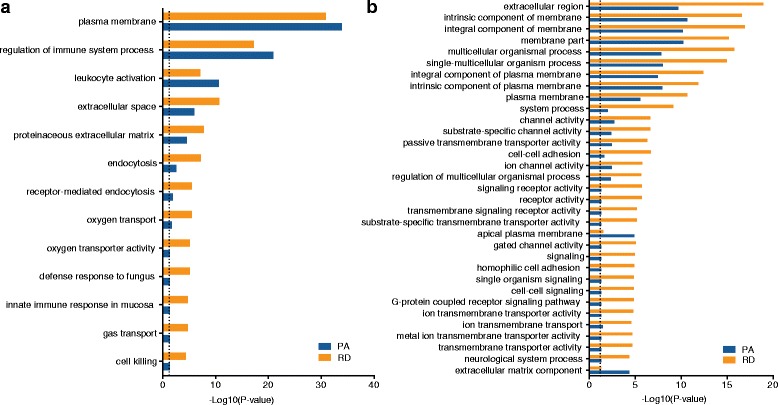
Pathway analysis. The GOrilla analyses show the statistically enriched (FDR ≤ 0.05) gene ontology terms in ascending (**a**) and descending orders (**b**). The RD method shows a greater number of significant enrichments as compared to the PA enrichment method. The dotted line shows the adjusted *p* value (FDR ≤ 0.05) cut-offs in both comparisons

We also sought to compare the effect of library preparation methods on pathway enrichment analysis through the use of the GOrilla threshold free enrichment approach [38] and Gene Ontology [39]. For this analysis, protein-coding genes were ranked according to their fold-change in an AML sample compared to its protocol-matched ALL sample and the whole gene list was analyzed in both ascending (Fig. 7a) and descending (Fig. 7b) order. Overall, the GOrilla analyses resulted in similar sets of statistically enriched (FDR ≤ 0.05) gene ontology terms. Interestingly, analysis of the RD data resulted in slightly more significant enrichments (Fig. 7a and b), an indication of a stronger signal and consistency across the different gene lists. However, in most cases and for both protocols, the reported pathways were rather generic and revealed no knowledge on disease pathogenesis. Additional pathway enrichment analysis was performed using QIAGEN’s Ingenuity® Pathway Analysis (IPA®, QIAGEN Redwood City, https://www.qiagenbioinformatics.com/products/ingenuity-pathway-analysis/). This revealed 19 and 16 canonical pathways enriched by genes differentially expressed between protocol-matched AML and ALL samples in the PA and RD comparisons, respectively (Additional file 1 and Additional file 2: Table S7).

## Discussion

With the unique ability to comprehensively characterize transcriptomes, RNA-seq has the potential to revolutionize clinical testing for a wide range of diseases. However, before the broader translation of RNA-seq into clinical practice, additional knowledge is needed to guide the selection of the library preparation protocol, as different alternatives can have a significant effect on downstream analysis and interpretation of RNA-seq outputs [12, 15, 18]. The performance of RNA-seq library preparation protocols has mostly been tested on non-clinical samples [16, 18–20] and the impact of protocols on clinical decision-making has not yet been addressed systematically. Some studies have even reported marked inconsistencies between RNA-seq data originating from differently processed libraries [19, 38], indicating that RNA-seq library preparation could also influence clinical decision-making.

To fill the gap in knowledge and assess the role of library preparation protocols on the detection of clinically important molecular characteristics, we applied two mainstream library preparation protocols to samples from leukemia patients and systematically compared their performance in a precision medicine setting. Although the small number of patient samples in our study is a limitation, especially for differential gene expression and pathway analysis, it absolutely mimics the clinical scenario and provides a fair equivalent to the current personalized medicine practices. Also, it is important to study the behavior of RNA-seq in a small-*n* setting to understand how RNA-seq performs in situations where only a few samples are available with no possibility of multiple replicates. Otherwise there is a risk that RNA-seq protocols are evaluated using metrics non-optimal for the goals of precision medicine and a wrong protocol is translated to standard clinical practice. Importantly, our study highlighted important differences between RNA-seq protocols, some of which were even clinically relevant, and indicated that that library preparation protocols have differing preferences for differential gene expression analysis, transcriptome characterization, fusion gene detection, and variant discovery.

Read and read mapping statistics demonstrated PA and RD libraries were largely comparable. All libraries were constructed from high quality RNA, had approximately similar insert sizes, and contained roughly the same amount of different types of reads. In line with recently published studies [12, 18], the PA protocol captured more transcripts emanating from exonic regions than the RD protocol. Given that these exon-mapping reads positively affect differential expression analysis [40], the PA protocol is the preferred method in understanding differential expression. In contrast, the higher intergenic or intronic mapped reads counts in the RD protocol can be advantageous in understanding pre-mRNA dynamics and identifying previously uncharacterized transcripts [17]. Moreover, the RD protocol captured 6.3% more non-coding RNAs compared to the PA protocol and depleted less non-coding RNAs in our qRT-PCR results suggesting its superiority at characterizing the non-coding RNA landscapes of the leukemia samples. In agreement with Sultan et al., [15] we found more rRNA mapping reads in the PA libraries than RD libraries, suggesting higher efficiency of the RD method to remove rRNAs compared to the PA method. This finding was further supported by expression estimation of rRNA genes in two patient samples by qRT-PCR. However, the rRNA mapping rates varied highly between technical replicate libraries, indicating that this step was greatly affected by experiment and preparation-dependent factors.

Regarding protein coding gene identification, the RD protocol performed a bit better and captured a wider repertoire. This method, for example, detected many non-polyadenylated protein-coding genes missed by the PA protocol, corroborating results from an earlier study [15]. Despite the better capture efficiency of the RD protocol, several known oncogenes were missed by this method. For example, *TGF-β1*, *BCL3* and *BRD4*, which all have been linked with leukemia development [23–25] were overlooked by the RD protocol. This suggests that the PA protocol may suit better for characterization of leukemia transcriptomes and indicates that selection of the RNA-seq library protocol should be guided by the objectives of the study.

Protein coding transcript abundances were largely in agreement and a high level of concordance was found between the RPKMs of matched PA and RD samples. If extrapolated to other leukemia studies, our results indicate that biological features impact more on RNA-seq data reproducibility than library preparation. Moreover, disease heterogeneity rather than the protocol limits the comparison of RNA-seq data across leukemia studies. Nevertheless, the PA protocol quantified expression differences between AML and ALL better and provided constantly higher RPKM values for leukemia marker genes. This implies the suitability of the PA protocol for differential expression analysis is partly attributed to the higher number of exon-mapping reads in PA libraries, indicating that the read depth should be a key consideration in the adaption of RNA-seq to leukemia samples.

Validation by qRT-PCR also highlighted the suitability of the PA protocol for gene expression analysis. In particular, the PA protocol detected mRNAs efficiently and more accurately reproduced expression differences in clinical samples. Moreover, the PA protocol performed better in the analysis of breast cancer cell lines, emphasizing that the effect of the library preparation is consistent irrespective of the source of RNA material and type of cancer. In contrast, the RD protocol efficiently depleted rRNA molecules compared to the PA protocol and was better suited for the non-coding RNA detection. Overall, the qRT-PCR results suggested that PA better mimics total RNA when analyzing mRNA transcripts, which could be explained by the higher efficiency of the qRT-PCR reaction in PA libraries and presence of mature mRNAs in this RNA preparation.

Abnormal fusion genes caused by chromosomal rearrangement are important genomic events in leukemia and characterize a substantial population of the leukemia cases. For example, the *BCR*-*ABL1* fusion gene is detected in 25–30% of young adult ALL cases [41] and is a clinical marker for treatment with targeted drugs. Markedly, both RNA-seq protocols successfully identified all known clinical diagnostic fusion genes *BCR*-*ABL1*, *MLLT4*-*MLL* and *TCF3*-*PBX1* in the patient material with high numbers of supporting reads. This indicates that RNA-seq and fusion gene analysis can sensitively detect fusions despite some previous claims to the contrary [42]. In addition, many potentially false positive predictions were made and fusion genes were called rather discordantly even between protocol and technical replicates. However, most of the discordantly detected gene fusions were supported only by a small number of spanning read pairs, indicating that precision could be improved significantly using stricter filtering parameters. Results from PCR amplification of low confidence fusion genes supported by <10 reads also suggest that fusion genes supported by few spanning reads may be false positives and should be validated by other methods, if these fusions are of interest. However, validation methods such as PCR amplification followed by Sanger sequencing may not be sensitive to detect low expressed fusions.

## Conclusion

Overall, this comparative study provides preliminary guidelines for the use of RNA-seq in a personalized medicine and other small-*n* setting, especially for hematological malignancies. In general, it showed that both PA and RD protocols produced consistent measures and were largely of similar usability. However, the PA protocol outperformed the RD protocol in more tests and it showed improved performance in gene expression analysis, classification of leukemia patients, quantification of leukemic marker genes, and variant analysis, which are all important for clinical sample assessment. Given that the study included only a limited number replicates, it would be beneficial to validate results using a larger cohort. Additionally, the effect of cDNA synthesis on library composition should be evaluated.

## Additional files

Additional file 1: Detailed descriptions of the data analysis methods and tools are provided. (DOCX 35 kb)

Additional file 2: **Table S1.** Biodetection analysis table generated using NOISeq package. **Table S2.** The list of genes showing differential expression in RD and PA methods based on clustering of overall gene expression data. **Table S3.** The list of genes and drugs used for ROC analysis. **Table S4.** Phi-correlation-variant calling. **Table S5.** GATK RNA-seq variant calling and variant annotation pipeline statistics. **Table S6.** Recurrent mutations identified by GATK. **Table S7.** Pathway analysis. **Table S8.** The sequences for primers used in qRT-PCR and fusion gene PCR amplification experiments. **Table S9.** PCR and Sanger sequencing details for fusion gene validation. (XLS 208 kb)

Additional file 3: **Figure S1.** Gene body coverage showing average coverage on the X-axis and percentile of gene body (5′- > 3′) on Y-axis in all leukemia patient samples. **Figure S2.** Overlapping genes among leukemia patient samples with RPKM >0.125. **Figure S3.** Overlapping protein coding genes among leukemia patient samples involved in library comparison analysis with RPKM >0.125. **Figure S4.** Overlapping protein coding genes in technical replicates of leukemia patient samples RPKM >0.125. **Figure S5.** The percentage of targeted bases covered at 5×, 10×, 15×, 20×, 25× and 30× depths. **Figure S6.** Rank correlation of RPKM values among gene expression profiles of patient samples. **Figure S7.** qRT-PCR validation in patient samples, log2-fold change expression of 5 genes in two leukemia patient samples (ALL 542 and AML 800) using PA and RD protocols compared to total RNA shows the PA protocol prepared RNA is closer to total RNA. In case of the PA protocol, only one gene *NABP1* shows significant difference compared to total RNA. On the other hand, three genes *POLR1B*, *SRM*, *TGFB1* show significant differences in expression. **Figure S8.** Fusion genes detected by the FusionCatcher tool. (PDF 2580 kb)
