# Supplementary material for: The impact of RNA sequence library construction protocols on transcriptomic profiling of leukemia
Source: BMC Genomics. 2017 Aug 17;18:629. doi: 10.1186/s12864-017-4039-1 (PMC5561555; doi:10.1186/s12864-017-4039-1)
Supplement: Supplementary file 1 — Detailed descriptions of the data analysis methods and tools are provided. (DOCX 35 kb) [file 12864_2017_4039_MOESM1_ESM.docx]

**The impact of RNA sequence library construction protocols on transcriptomic profiling of leukemia**

Ashwini Kumar,^1^ Matti Kankainen,^1,2^ Alun Parsons,^1^ Olli Kallioniemi,^1,3^ Pirkko Mattila,^1,4^ Caroline A. Heckman^1^

Affiliations:

^1^Institute for Molecular Medicine Finland (FIMM), Helsinki Institute of Life Science, University of Helsinki, Helsinki, Finland

^2^Medical and Clinical Genetics, University of Helsinki and Helsinki University Hospital, Helsinki, Finland

^3^Science for Life Laboratory, Karolinska Institutet, Solna, Sweden

^4^Finnish Red Cross Blood Service, Kivihaantie 7, Helsinki, Finland

**DATA ANALYSIS**

**Read pre-processing and alignment**

Trimmomatic version 0.32 [1] was used to correct sequence data for Illumina adaptor sequences (TruSeq3-PE.fa:2:30:10), trailing bases with quality below 3 (LEADING:3 and TRAILING:3), low quality segments with an average quality in a 4-base wide sliding below 15 (SLIDINGWINDOW:4:15), and reads less than 36-bp in length after trimming (MINLEN:36). The spliced alignment of the processed paired-end reads was then performed against the human reference genome (Ensembl GRCh38) with the guidance of the EnsEMBL reference gene models (EnsEMBL v80) using the gap-aware STAR aligner [2] (ver. 2.3.0). The default 2-pass per-sample alignment and indexing settings were used, except that the overhang on each side of a splice junction was set to 99. Picard tool version 1.63 was used to add read groups, mark duplicates, and sort reads in the resulting alignment files.

**Quality check**

Quality control analysis was performed using FastQC version v0.11.5 [10] and RNA-SeQC version 1.1.8 [3] with BWA [4] version 0.5.10. The default settings were used, except that RNA-SeQC was used with an EnsEMBL v80 reference annotation file and a custom GC content file downloaded from Biomart [5]. In addition, we used a custom rRNA sequence index including human rRNA elements from i) the EnsEMBL genome annotation file (elements with gene or transcript bioptype rRNA or Mt_rRNA); ii) RFAM [6] (human rRNA sequences of which name matched terms ribosomal RNA and 5.8S, 5S, large or small); and iii) the SILVA database [7] (human elements listed in the LSURef and SSURef databases).

**Read counts, RPKM and CPM calculation, and biodetection plots**

The RSubread version 1.14.2 [8] was employed for read summarization from aligned bam files using the same genome annotation file that was used in the reference read alignment and quality control (EnsEMBL v80). Default parameters were used to count the number of reads aligned to each gene, except that a read was assigned to more than one feature (or meta-feature) if it overlapped with more than one feature (or meta-feature). RPKM and CPM were calculated using edgeR package [9] version 3.12.0. RSeQC [10] version 2.6.3 was used to generate gene body coverage plots with default parameters. Biodetection plot matrices were generated using NOIseq [11] version 2.6.0 with the default settings.

**ROC curve analysis**

ROC curves were computed using R version 3.2.4. In this analysis, different absolute or fold-change gene expressions were used to categorize genes into true and false positives and negatives. Altogether, six ROC curves (Figure 3a-f) were generated. For the ROC analysis, genes were selected from published studies on leukemia gene expression (Additional file 3: Table S3).

1. The genes used in Figure 3a were selected from the leukemia specific gene expression study by Haferlach at el [12], which describes a global approach for the diagnosis of leukemia using gene expression profiling. The study reported 13 lists of genes with each containing 100 genes from one-versus-all leukemia type comparisons. In our study, AML and ALL specific genes (n=421) were selected for ROC analysis with unique Ensembl gene identifiers.
2. The genes used in Figure 3b were selected from three published studies that reported differentially expressed genes between AML and ALL. A) Armstrong and colleagues [13] reported the top 15 genes, which highly correlated with AML as compared to ALL. B) Thomas et al [14] compared the expression profiles of 11 AML and 27 ALL samples and reported 25 genes in AML and 25 genes in ALL as highly expressed. C) Kohlman at el [15] reported gene expression signatures in 90 leukemia patient samples and reported a minimal set of informative genes for classification of AML and ALL subtypes. In total, we collected 78 genes for ROC analysis.
3. The gene targets were calculated using KiBA [16] analysis for AML and ALL clinically used drugs. KiBA analysis reported 142 unique gene targets for 17 of the most frequently used clinical drugs (Additional file 3: Table S3) (Figure 3c).
4. Drug sensitivity and resistance testing was performed in-house with AML and ALL patient cases considered in this study [17]. The gene targets were calculated using KiBA analysis for the top sensitive and resistant drugs based on the drug testing results (Additional file 3: Table S3). The expressions of these target genes were used in the ROC analysis (Figure 3d).
5. Microarray Innovations in LEukemia (MILE) study stage 2 cohort data [18] represent data on a retrospective whole-genome analysis phase. The custom microarray dataset had 1400 leukemia disease specific genes from 1,152 acute and chronic leukemia patients. The probe identifiers for more than one gene were removed and unique probe ids were selected (925 including 4 controls). We used the RPKM of these selected genes to generate the ROC plot (Figure 3e).
6. Each leukemia type has a characteristic set of cluster of differentiation (CD) marker genes that facilitates the classification and diagnosis of hematopoietic malignancies [19]. Hence, expressions of all the CD marker genes (n = 98) were selected for the ROC analysis (Figure 3f).

**Fusion gene detection**

FusionCatcher version 0.99.4c [20] was used to detect expressed fusion genes using default parameters. FusionCatcher was applied to raw, un-processed reads (FASTQ files).

**Pathway analysis**

Pathway analysis was performed using QIAGEN’s Ingenuity® Pathway Analysis (IPA®, QIAGEN Redwood City, [www.qiagen.com/ingenuity)](http://www.qiagen.com/ingenuity)). The method was applied to protein-coding genes (defined based on their Ensembl biotype annotations) with ≥ 2-fold change in expression between protocol-matched AML and ALL samples. Z-scores > abs (2) was considered as significant. In addition to IPA, the GOrilla [21] web-server was used to identify enriched terms across all three-gene ontologies. For analysis, protein-coding genes were ranked according to their fold-change in an AML sample compared to its protocol-matched ALL sample. Both descending and ascending ordered gene lists were analyzed with default settings.

**Additional pathway enrichment analysis**

Additional pathway enrichment analyses were performed using QIAGEN’s Ingenuity® “Pathway Analysis (IPA®, QIAGEN Redwood City, [www.qiagen.com/ingenuity)](http://www.qiagen.com/ingenuity))” by analyzing protein-coding genes with at least a two-fold change in expression. Overall, the analysis revealed 19 and 16 canonical pathways enriched by genes differentially expressed between protocol-matched AML and ALL samples in the PA and RD comparisons, respectively (Additional file 3: Table S7). While most of the identified pathways were without obvious clinical and medical relevance, a few pathways relevant to tumorigenesis were also discovered in the analysis. Among these were the NF-κB pathway that was identified by both comparisons, as well as PI3K signaling in T lymphocytes and Jak-Stat pathways that were discovered only based on the PA data.

**Variant calling**

Expressed variants were called using the GATK Best Practice [22] for variant calling on RNA-seq using default parameters. Specifically, pre-processed and mapped reads were split into exon segments using GATK SplitNCigarReads, local indel realignment was performed around indels using GATK IndelRealigner, and base qualities were recalibrated using GATK BaseQualityScoreRecalibration module. Variant calls were identified with the GATK HaplotypeCaller and filtered using GATK VariantFiltration tool according to the best practice recommendations for the RNA-seq variant analysis workflow.

Variant annotation was done by the ANNOVAR [23] software against RefSeq gene models [24]. In this process, we retained non-synonymous, stop-loss, stop-gain, and frameshift variants with a genotype quality score ≥ 40, read depth ≥ 10, and minor allele frequency < 0.5%. The minor allele frequencies were derived from the NHLBI exome sequencing (ESP) 6500 [25], 1000 Genomes [26], and Exome Aggregation Consortium (ExAC) [27] databases. Matching of discovered variants for AML and ALL associated nonsense (n = 743 bp), missense (n = 12782 bp), frameshift deletion (2044 bp), and frameshift insertion (1047 bp) was done using the COSMIC database [28] based on genomic coordinates.

**REFERENCES**

1. Bolger A, Giorgi F: Trimmomatic: a flexible read trimming tool for illumina NGS data. *URL* [*http://www.usadellab.org/cms/index.php*](http://www.usadellab.org/cms/index.php) 2014, **;**.

2. Dobin A, Davis CA, Schlesinger F, Drenkow J, Zaleski C, Jha S, Batut P, Chaisson M, Gingeras TR: STAR: ultrafast universal RNA-seq aligner. *Bioinformatics* 2013, 29(1)**;**15-21.

3. DeLuca DS, Levin JZ, Sivachenko A, Fennell T, Nazaire MD, Williams C, Reich M, Winckler W, Getz G: RNA-SeQC: RNA-seq metrics for quality control and process optimization. *Bioinformatics* 2012, 28(11)**;**1530-1532.

4. Li H, Durbin R: Fast and accurate short read alignment with Burrows-Wheeler transform. *Bioinformatics* 2009, 25(14)**;**1754-1760.

5. Kinsella RJ, Kähäri A, Haider S, Zamora J, Proctor G, Spudich G, Almeida-King J, Staines D, Derwent P, Kerhornou A: Ensembl BioMarts: a hub for data retrieval across taxonomic space. *Database* 2011, 2011**;**.

6. Gardner PP, Daub J, Tate JG, Nawrocki EP, Kolbe DL, Lindgreen S, Wilkinson AC, Finn RD, Griffiths-Jones S, Eddy SR, Bateman A: Rfam: updates to the RNA families database. *Nucleic Acids Res* 2009, 37(Database issue)**;**D136-40.

7. Quast C, Pruesse E, Yilmaz P, Gerken J, Schweer T, Yarza P, Peplies J, Glockner FO: The SILVA ribosomal RNA gene database project: improved data processing and web-based tools. *Nucleic Acids Res* 2013, 41(Database issue)**;**D590-6.

8. Liao Y, Smyth GK, Shi W: The Subread aligner: fast, accurate and scalable read mapping by seed-and-vote. *Nucleic Acids Res* 2013, 41(10)**;**e108.

9. Robinson MD, McCarthy DJ, Smyth GK: edgeR: a Bioconductor package for differential expression analysis of digital gene expression data. *Bioinformatics* 2010, 26(1)**;**139-140.

10. Wang L, Wang S, Li W: RSeQC: quality control of RNA-seq experiments. *Bioinformatics* 2012, 28(16)**;**2184-2185.

11. Tarazona S, García F, Ferrer A, Dopazo J, Conesa A: NOIseq: a RNA-seq differential expression method robust for sequencing depth biases. *EMBnet.journal* 2012, 17(B)**;**pp. 18-19.

12. Haferlach T, Kohlmann A, Schnittger S, Dugas M, Hiddemann W, Kern W, Schoch C: Global approach to the diagnosis of leukemia using gene expression profiling. *Blood* 2005, 106(4)**;**1189-1198.

13. Armstrong SA, Staunton JE, Silverman LB, Pieters R, den Boer ML, Minden MD, Sallan SE, Lander ES, Golub TR, Korsmeyer SJ: MLL translocations specify a distinct gene expression profile that distinguishes a unique leukemia. *Nat Genet* 2002, 30(1)**;**41-47.

14. Thomas JG, Olson JM, Tapscott SJ, Zhao LP: An efficient and robust statistical modeling approach to discover differentially expressed genes using genomic expression profiles. *Genome Res* 2001, 11(7)**;**1227-1236.

15. Kohlmann A, Schoch C, Schnittger S, Dugas M, Hiddemann W, Kern W, Haferlach T: Molecular characterization of acute leukemias by use of microarray technology. *Genes Chromosomes Cancer* 2003, 37(4)**;**396-405.

16. Tang J, Szwajda A, Shakyawar S, Xu T, Hintsanen P, Wennerberg K, Aittokallio T: Making sense of large-scale kinase inhibitor bioactivity data sets: a comparative and integrative analysis. *J Chem Inf Model* 2014, 54(3)**;**735-743.

17. Pemovska T, Kontro M, Yadav B, Edgren H, Eldfors S, Szwajda A, Almusa H, Bespalov MM, Ellonen P, Elonen E, Gjertsen BT, Karjalainen R, Kulesskiy E, Lagstrom S, Lehto A, Lepisto M, Lundan T, Majumder MM, Marti JM, Mattila P, Murumagi A, Mustjoki S, Palva A, Parsons A, Pirttinen T, Ramet ME, Suvela M, Turunen L, Vastrik I, Wolf M, Knowles J, Aittokallio T, Heckman CA, Porkka K, Kallioniemi O, Wennerberg K: Individualized systems medicine strategy to tailor treatments for patients with chemorefractory acute myeloid leukemia. *Cancer Discov* 2013, 3(12)**;**1416-1429.

18. Haferlach T, Kohlmann A, Wieczorek L, Basso G, Kronnie GT, Bene MC, De Vos J, Hernandez JM, Hofmann WK, Mills KI, Gilkes A, Chiaretti S, Shurtleff SA, Kipps TJ, Rassenti LZ, Yeoh AE, Papenhausen PR, Liu WM, Williams PM, Foa R: Clinical utility of microarray-based gene expression profiling in the diagnosis and subclassification of leukemia: report from the International Microarray Innovations in Leukemia Study Group. *J Clin Oncol* 2010, 28(15)**;**2529-2537.

19. Lewis RE, Cruse JM, Sanders CM, Webb RN, Suggs JL: Aberrant expression of T-cell markers in acute myeloid leukemia. *Exp Mol Pathol* 2007, 83(3)**;**462-463.

20. Nicorici D, Satalan M, Edgren H, Kangaspeska S, Murumagi A, Kallioniemi O, Virtanen S, Kilkku O: FusionCatcher-a tool for finding somatic fusion genes in paired-end RNA-sequencing data. *bioRxiv* 2014, **;**011650.

21. Eden E, Navon R, Steinfeld I, Lipson D, Yakhini Z: GOrilla: a tool for discovery and visualization of enriched GO terms in ranked gene lists. *BMC Bioinformatics* 2009, 10**;**48-2105-10-48.

22. DePristo MA, Banks E, Poplin R, Garimella KV, Maguire JR, Hartl C, Philippakis AA, Del Angel G, Rivas MA, Hanna M: A framework for variation discovery and genotyping using next-generation DNA sequencing data. *Nat Genet* 2011, 43(5)**;**491-498.

23. Wang K, Li M, Hakonarson H: ANNOVAR: functional annotation of genetic variants from high-throughput sequencing data. *Nucleic Acids Res* 2010, 38(16)**;**e164.

24. O'Leary NA, Wright MW, Brister JR, Ciufo S, Haddad D, McVeigh R, Rajput B, Robbertse B, Smith-White B, Ako-Adjei D: Reference sequence (RefSeq) database at NCBI: current status, taxonomic expansion, and functional annotation. *Nucleic Acids Res* 2015, **;**gkv1189.

25. Tennessen JA, Bigham AW, O'Connor TD, Fu W, Kenny EE, Gravel S, McGee S, Do R, Liu X, Jun G, Kang HM, Jordan D, Leal SM, Gabriel S, Rieder MJ, Abecasis G, Altshuler D, Nickerson DA, Boerwinkle E, Sunyaev S, Bustamante CD, Bamshad MJ, Akey JM, Broad GO, Seattle GO, NHLBI Exome Sequencing Project: Evolution and functional impact of rare coding variation from deep sequencing of human exomes. *Science* 2012, 337(6090)**;**64-69.

26. Siva N: 1000 Genomes project. *Nat Biotechnol* 2008, 26(3)**;**256-256.

27. Lek M, Karczewski KJ, Minikel EV, Samocha KE, Banks E, Fennell T, O’Donnell-Luria AH, Ware JS, Hill AJ, Cummings BB: Analysis of protein-coding genetic variation in 60,706 humans. *Nature* 2016, 536(7616)**;**285-291.

28. Bamford S, Dawson E, Forbes S, Clements J, Pettett R, Dogan A, Flanagan A, Teague J, Futreal PA, Stratton MR: The COSMIC (Catalogue of Somatic Mutations in Cancer) database and website. *Br J Cancer* 2004, 91(2)**;**355-358.
