# Supplementary material for: The impact of RNA sequence library construction protocols on transcriptomic profiling of leukemia
Source: BMC Genomics. 2017 Aug 17;18:629. doi: 10.1186/s12864-017-4039-1 (PMC5561555; doi:10.1186/s12864-017-4039-1)

2. Supplementary Figures

Figure Legends:
Figure S1. Gene body coverage showing average coverage on the X-axis and percentile of gene body (5´-> 3´) on Y-axis in all leukemia patient samples.

Figure S2. Overlapping genes among leukemia patient samples involved in library comparison analysis with RPKM > 0.125 for this analysis.

Figure S3. Overlapping protein coding genes among leukemia patient samples involved in library comparison analysis with RPKM > 0.125 for this analysis.

Figure S4. Overlapping protein coding genes in technical replicates of leukemia patient samples involved in library comparison analysis with RPKM > 0.125 for this analysis.

Figure S5. The percentage of targeted bases covered at 5X, 10X, 15X, 20X, 25X and 30X depths.

Figure S6. Rank correlation of RPKM values among gene expression profiles of patient samples.

Figure S7. qRT-PCR validation in patient sample, log2 fold change expression of 5 genes in two leukaemia patient samples (ALL 542 and AML 800) using PA and RD protocols compared to total RNA, shows the PA protocol prepared RNA is closer to total RNA. In case of PA only one gene NABP1 shows significant difference compared to the total RNA. On the other hand, three genes POLR1B, SRM, TGFB1 shows significant difference in the expression.

Figure S8. Fusion genes detected by FusionCatcher tool.

Figure S1. Gene body coverage

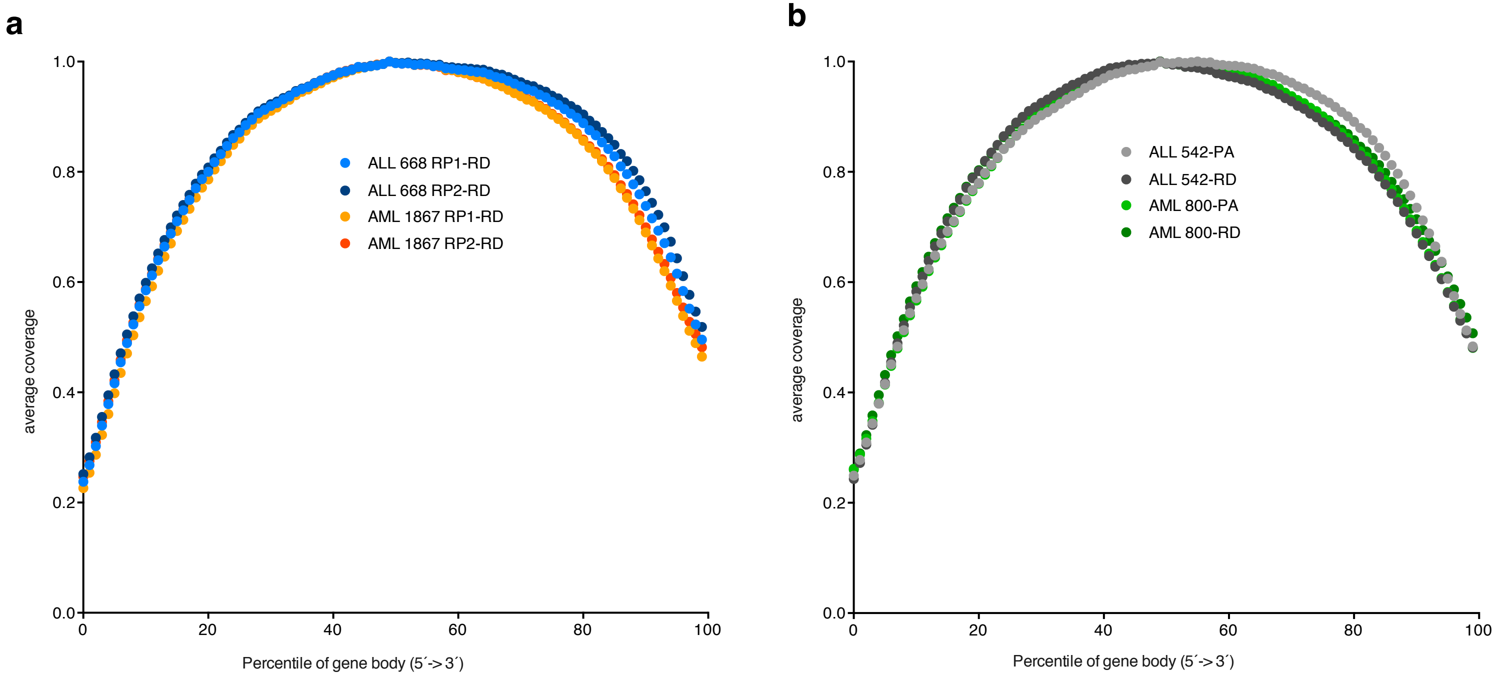

Figure S2. RPKM > 0.125 (all genes)

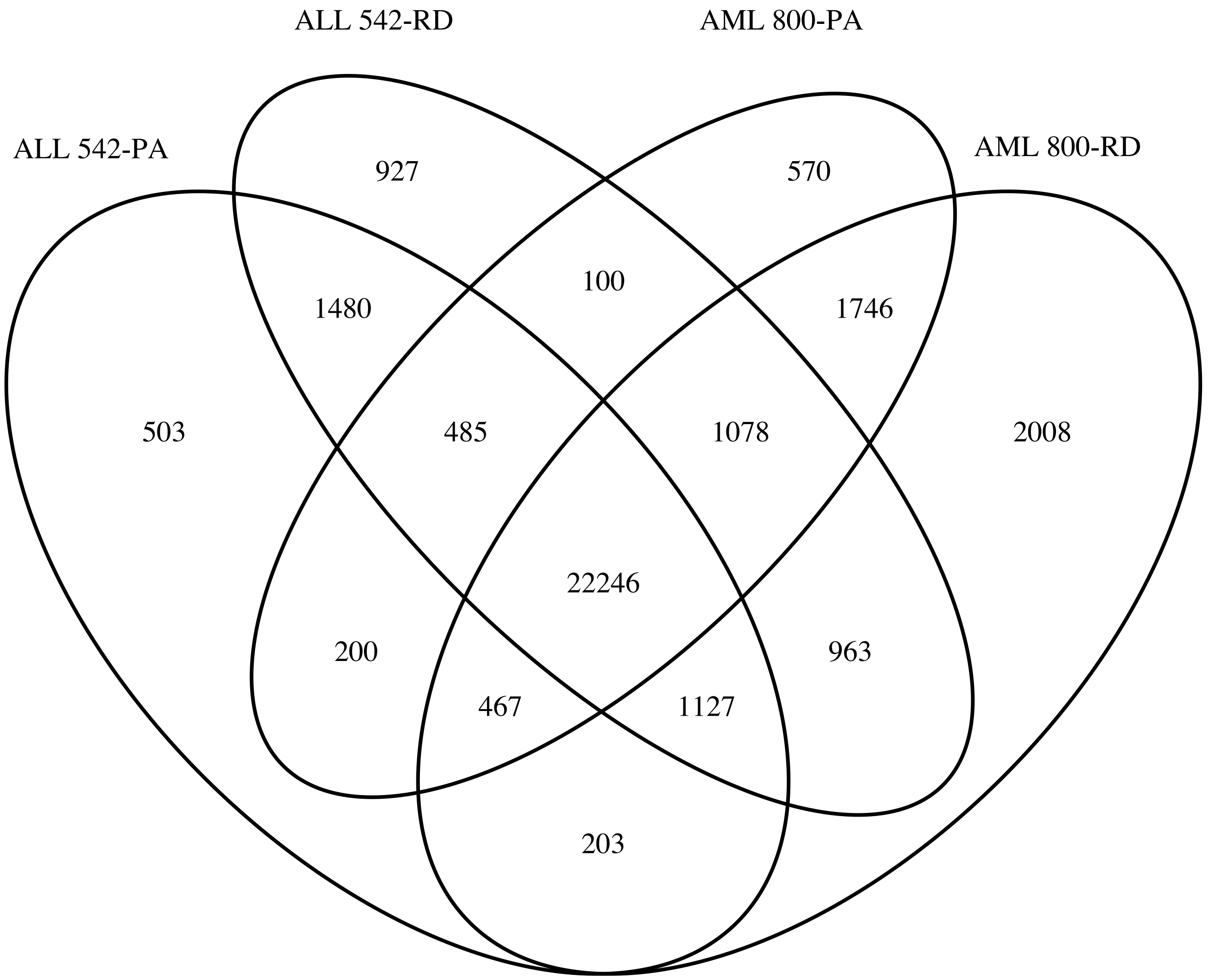

Figure S3. RPKM > 0.125 (Only protein coding genes)

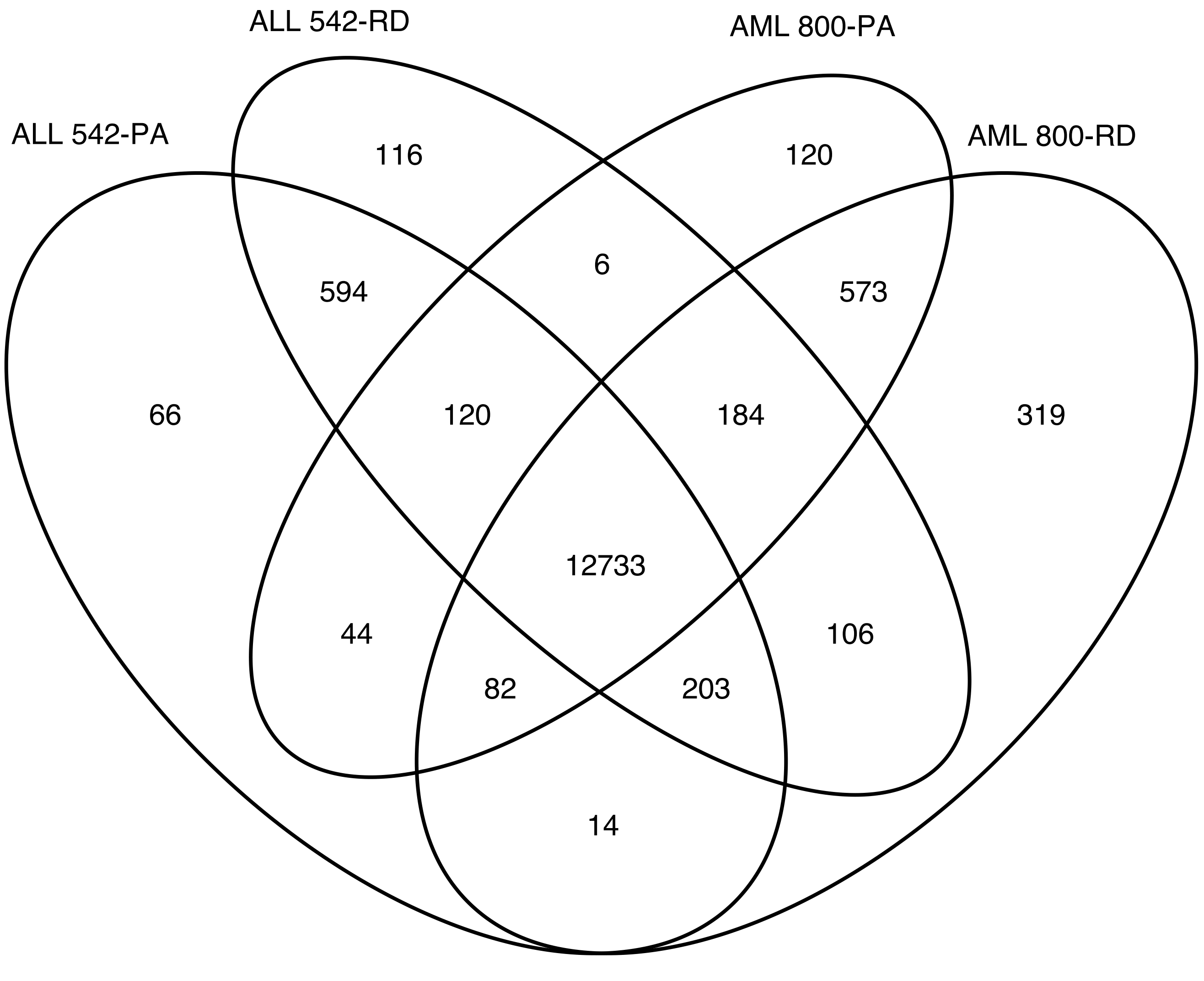

Figure S4. RPKM > 0.125 (Only protein coding genes)

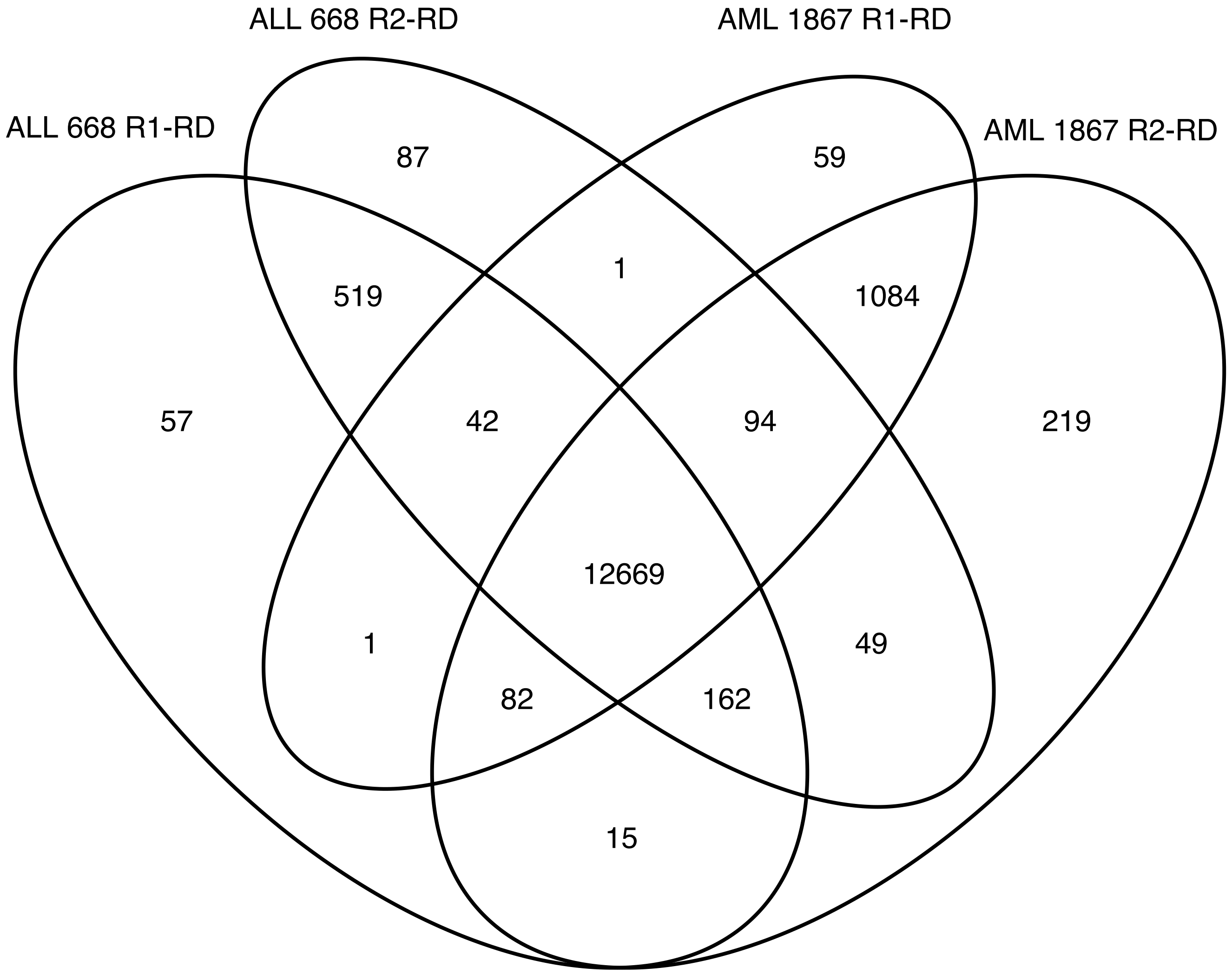

Figure S5. Cosmic coverage DP5-30

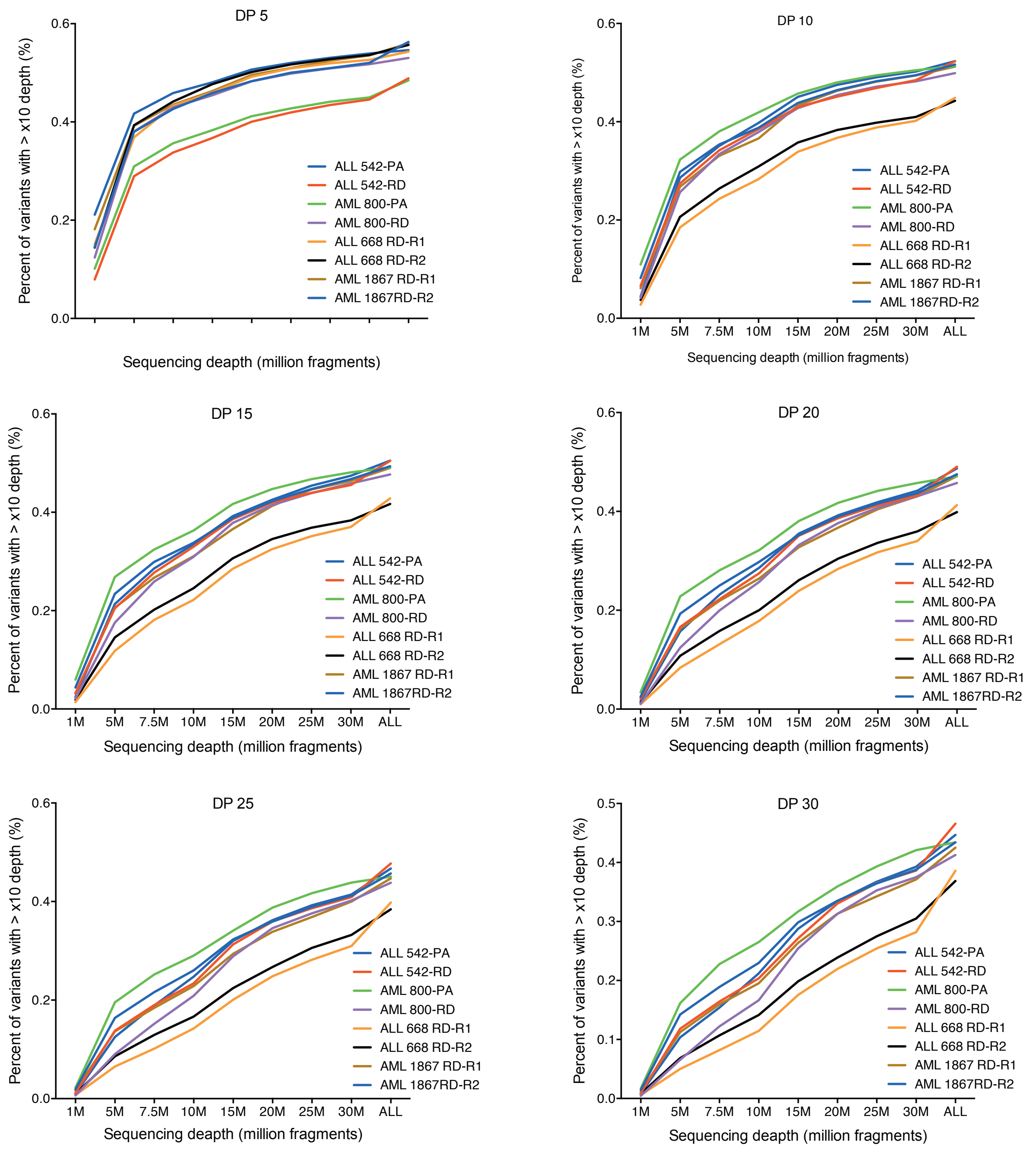

Figure S6. Rank correlation

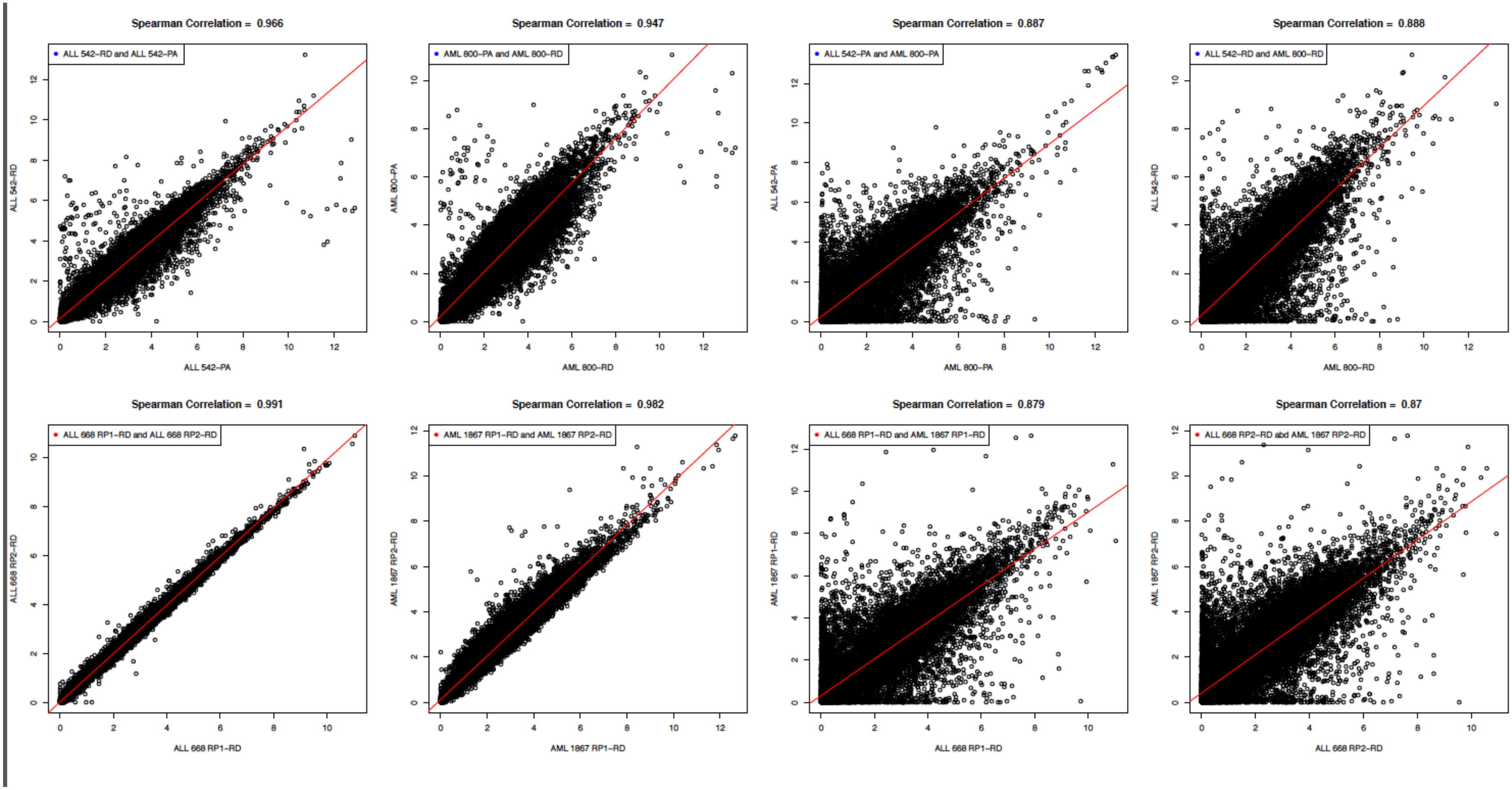

Figure S7. qRT-PCR validation in patient samples

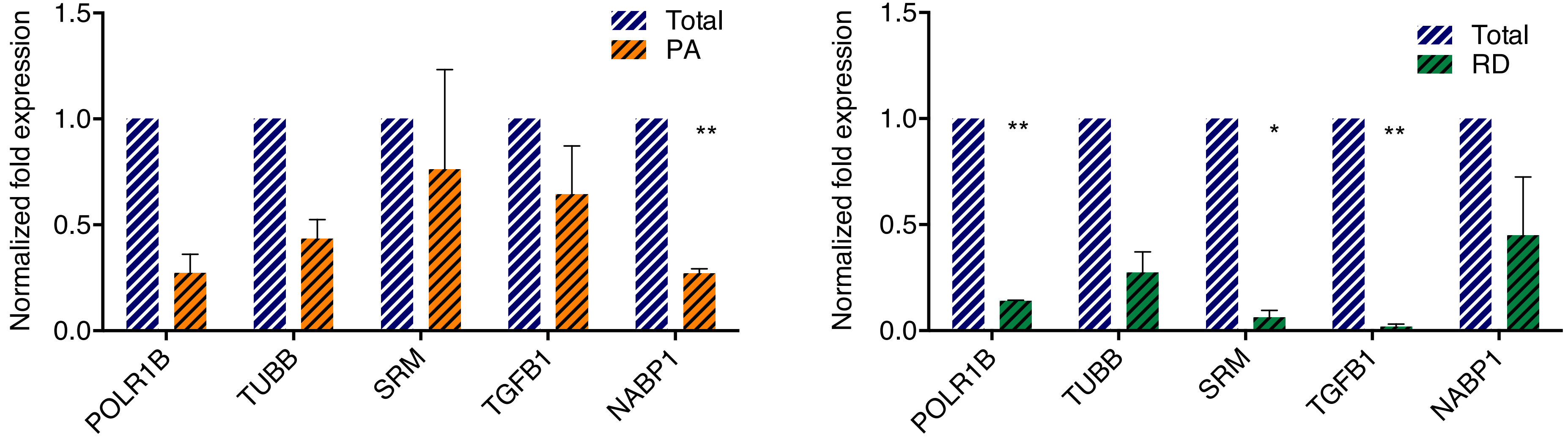

Figure S8. Detection of fusion genes

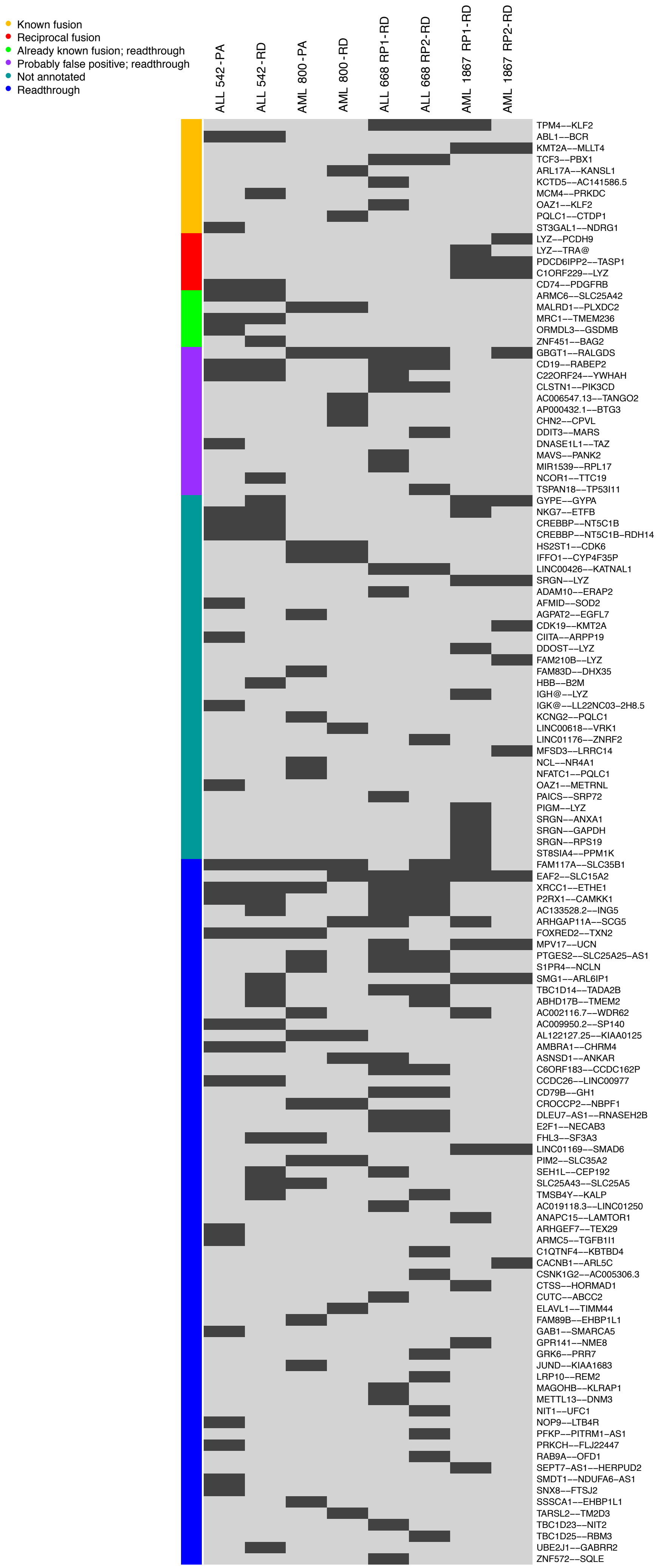

Supplement: Supplementary file 3 — Figure S1. Gene body coverage showing average coverage on the X-axis and percentile of gene body (5′- > 3′) on Y-axis in all leukemia patient samples. Figure S2. Overlapping genes among leukemia patient samples with RPKM >0.125. Figure S3. Overlapping protein coding genes among leukemia patient samples involved in library comparison analysis with RPKM >0.125. Figure S4. Overlapping protein coding genes in technical replicates of leukemia patient samples RPKM >0.125. Figure S5. The percentage of targeted bases covered at 5×, 10×, 15×, 20×, 25× and 30× depths. Figure S6. Rank correlation of RPKM values among gene expression profiles of patient samples. Figure S7. qRT-PCR validation in patient samples, log2-fold change expression of 5 genes in two leukemia patient samples (ALL 542 and AML 800) using PA and RD protocols compared to total RNA shows the PA protocol prepared RNA is closer to total RNA. In case of the PA protocol, only one gene NABP1 shows significant difference compared to total RNA. On the other hand, three genes POLR1B, SRM, TGFB1 show significant differences in expression. Figure S8. Fusion genes detected by the FusionCatcher tool. (PDF 2580 kb) [file 12864_2017_4039_MOESM3_ESM.pdf]
